# Controllable Fabrication of Au-Coated AFM Probes via a Wet-Chemistry Procedure

**DOI:** 10.1186/s11671-018-2789-6

**Published:** 2018-11-19

**Authors:** Lizhen Gao, Huiling Zhao, Yinli Li, Tianfeng Li, Dong Chen, Bo Liu

**Affiliations:** Institute of Photo-biophysics, School of Physics and Electronics, Henan University, Kaifeng, 475004 People’s Republic of China

**Keywords:** Tip-enhanced Raman spectrum, Wet-chemistry procedure, AFM-TERS, Strong enhancement effect

## Abstract

Tip-enhanced Raman spectroscopy (TERS), which offers a spatial resolution far beyond the limitations of the optical diffraction and detection sensitivity down to a single molecular level, has become one of the powerful techniques applied in current nanoscience and technology. However, the excellent performance of a TERS system is very much dependent on the quality of metallized probes used in TERS characterization. Thus, how to prepare higher-quality probes plays a vital role in the development and application of TERS technique. In this work, one simple wet-chemistry procedure was designed to fabricate atomic force microscopy-based TERS (AFM-TERS) probes. Through the controlled growth of a gold film on a commercial silicon AFM probe, TERS probes with different apex diameters were prepared successfully. A series of TERS results indicated that the probes with the apex size of 50~60 nm had the maximum TERS enhancement, and the Raman enhancement factor was in the range of 10^6^ to 10^7^. Compared with those prepared by other fabrication methods, our TERS probes fabricated by this wet-chemistry method have the virtues of good stability, high reproducibility, and strong enhancement effect.

## Introduction

Atomic force microscopy (AFM) has been widely applied in nanoscience for its high lateral resolution, simple operation, and environmental adaptability. In AFM, the surface information of a sample is acquired via the interaction force between the tip and sample, which is converted into the motion of a small spring-like cantilever with the tip at the end. The motion is detected by deflection of a semiconductor laser illuminating on the back of the AFM cantilever. If the tip (usually composed of silicon or silicon nitride) is covered with a metal layer and illuminated by a laser, the optical field enhancement surpassing the diffraction limit will be got because of the coefficient of surface plasmon resonance and lightning-rod effect [1–3]. Therefore, the topography and optical information of a sample can be characterized simultaneously with a nanoscale resolution when the metallized probe is scanning the sample surface. This is the principle of AFM-based tip-enhanced Raman spectroscopy (AFM-TERS). With high detection sensitivity and versatile characterization, AFM-TERS has been becoming a powerful tool for characterizing various materials, such as single molecules [4–7], biological materials [8–10], and low dimensional nanomaterials [11–13].

The probe is one of the key factors in AFM-TERS experiments because of its influence on the spatial resolution, reproducibility, and enhancement of the chemical information from the sample surface. Functional AFM-TERS probes are obtained by covering commercial probes of silicon or silicon nitride with silver (Ag) or gold (Au) layers owing to their strong surface plasmon resonance effect in the visible region and relatively high chemical stability compared with other metals [14–16]. Many methods have been developed to prepare metallized probes, among which vacuum evaporation is the most common method for TERS probe preparation because of its high efficiency and purity [17–19]. However, except for the disadvantage of low reproducibility, vacuum evaporation is recognized as an expensive method that requires relatively complex apparatus and a rigorous laboratory operating environment [20]. Therefore, commercial or homemade AFM-TERS probes fabricated by this method are both costly. Compared with vacuum deposition, chemical deposition has emerged as a nanofabrication method with the advantages of simple operation, environmental adaptability, and low cost [21–23]. However, further application of this technique has been hindered by the high surface roughness derived from low-density nucleation sites induced by the few defects on the smooth Si surface, as nucleation sites in chemical reactions are only formed on the defects of the probe.

To address this issue, in the present study, we took advantage of the principles of self-assembly and surface chemistry [24, 25]. Using a silanized probe rather than a smooth probe, a smooth metallic layer was successfully deposited onto AFM probe surfaces. The main change in the method was that the probe was silanized with thiol-terminal silanes before metal film deposition. With a proper reaction time, the coupling agent formed a homogeneous monolayer on the probe surface and acted as a primary reducing and stabilizing agent simultaneously. Thus, all sites on a probe surface were active sites because –SH could reduce Au^3+^ and adsorb it on the probe surface. The reaction equation is as follows [26]:

$$ 2{\mathrm{Au}}^{3+}+6\mathrm{RSH}=2{\mathrm{Au}}^0+3\mathrm{RSSR}+6{\mathrm{H}}^{+} $$

A series of characterization results indicated that Au layers were successfully deposited onto commercial AFM probe surfaces, and the diameters of tip apex could be effectively tuned through controlling experimental conditions. Au-coated AFM (Au@AFM) probes prepared by this wet-chemistry method were ideal optical probes for a 633-nm laser, exhibiting strong and highly tunable localized surface plasmon resonances in red visible light range.

## Methods

### Tips Silanization

Commercial Si AFM probe (VIT_P, NT-MDT Co., Moscow, Russia) was ozone cleaned for 30 s to render them hydroxylated, and then the probe was immersed in a 0.25-mM 3-merraptnpropylt rimethnxysilane (MPTS, 95%, Sigma-Aldrich) methanol solution for 30 min. After that, the probe was sequentially rinsed with chloroform, acetone, and ultrapure water to remove physisorbed ions and molecules on the probe surfaces. Finally, the probes were dried with nitrogen gas.

### Gold Film Growths

MPTS and sodium borohydride (NaBH_4_, 99%) were employed as the primary and secondary reducing agents, respectively, during deposition of an Au layer onto Si probe surfaces. In addition, MPTS also acted as a stabilizing agent for the Au layer. Gold layer was grown by immersing a silanized probe into 1.0% HAuCl_4_·3H_2_O (99%) aqueous solution for a few minutes, during which the AuCl_4_^−^ was reduced to Au^0^ and adsorbed onto the probe surface by S–Au bond. Therefore, the probe was withdrawn from HAuCl_4_ aqueous solution; its surface was covered with S–Au bonds and the aqueous film consisting of AuCl_4_^−^ ions. Then, it was dipped into a 1.0% NaBH_4_ aqueous solution to reduce the surplus AuCl_4_^−^ ions on the probe surface. Finally, the probe was cleaned with ultrapure water and dried with nitrogen gas. In this stage, the different diameter of probe apex was obtained through varying the immersion time or the number of immersion cycles. In our control experiments, the minimum immersion time was 5 min, and immersion cycles varied from 1 to 6, respectively.

### Performance Characterization

Morphological characterizations of the probes before and after depositing Au layers were performed using scanning electron microscopy (SEM, JEOL JSM-7001F, FEI). An NT-MDT Ntegra Raman/AFM system was used for TERS measurements to evaluate the Raman enhancement effects of these fabricated AFM-TERS probes. The instrument is equipped with a × 100 objective (N.A. = 0.7) and 633-nm laser excitation line. In addition, samples for TERS were provided with a Nile blue (NB) monolayer which was prepared by spin coating 10 μL of 5 × 10^−5^ M NB methanol solution onto a commercial Au-coated Si wafer [1].

## Results and Discussion

### SEM Image

**Fig. 1:**
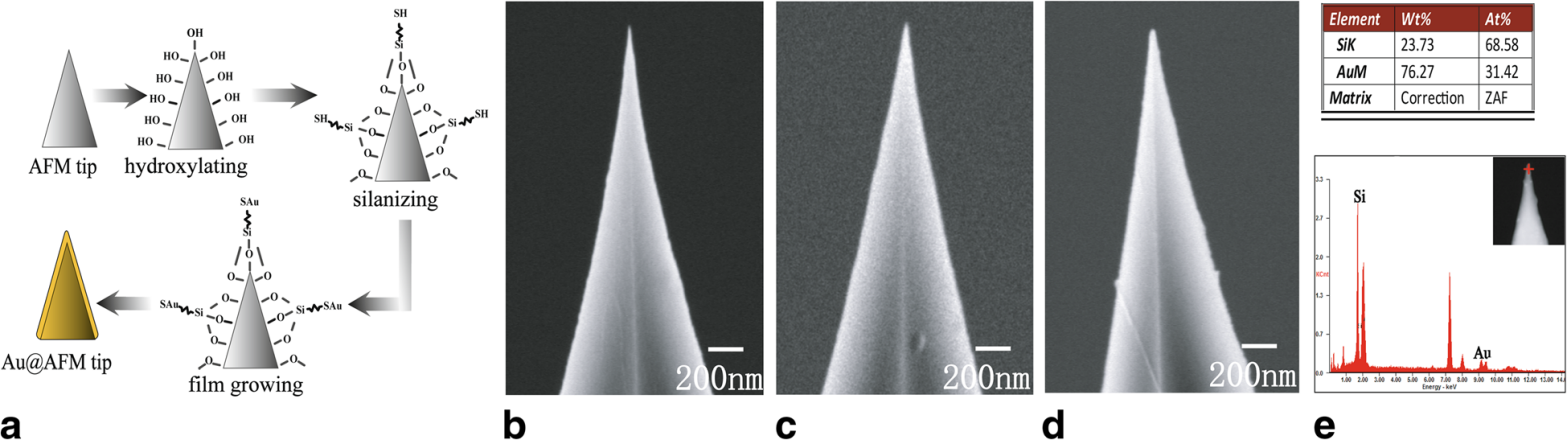
**a** Illustration of the wet-chemical procedure for preparing an AFM-based tip-enhanced Raman spectroscopy (AFM-TERS) probe. **b** A commercial Si AFM probe with the apex size < 15 nm. **c** Silanized probe with the apex diameter of ~ 20 nm. **d** Au@AFM probe after a 5 min immersion and apex diameter of 25 nm. **e** Energy dispersive spectrometer (EDS) of the probe in **d**

The fabrication process of Au@AFM probe was shown in Fig. 1a. Firstly, commercial Si AFM probe was hydroxylated by ozone. Next, the hydrophilic probe was immersed into an MPTS methanol solution to make the surface of the probe covered with –SH. Then, the silanized probe was dipped into the HAuCl_4_ aqueous solution for some time. At last, the probe was taken out and immerse into the NaBH_4_ aqueous solution to reduce surplus AuCl_4_^−^ and form Au film on the surfaces of the probes. SEM characterization before and after Au film deposition was carried out to observe the changing diameter of tip apex of AFM probes (Fig. 1b–d). Figure 1c revealed that the apex diameter of commercial Si AFM probe was slightly increased to ~ 20 nm after silanization procedure. In Fig. 1d, the tip apex diameter of one as-prepared Au@AFM probe was even about 25 nm. As no other material was introduced in this experiment, the size increase in probe apex could be ascribed to the growth of Au film on the probe surface. Regarding the coating composition on the tip surface, more evidence regarding the probe coating composition was collected by energy dispersive spectrometer (EDS). The results in Fig. 1e showed that the Au At% on the probe apex was 31.42% (Fig. 1e), which indicated that some Au atoms were deposited on the probe surface, but the amount was very little.

**Fig. 2:**
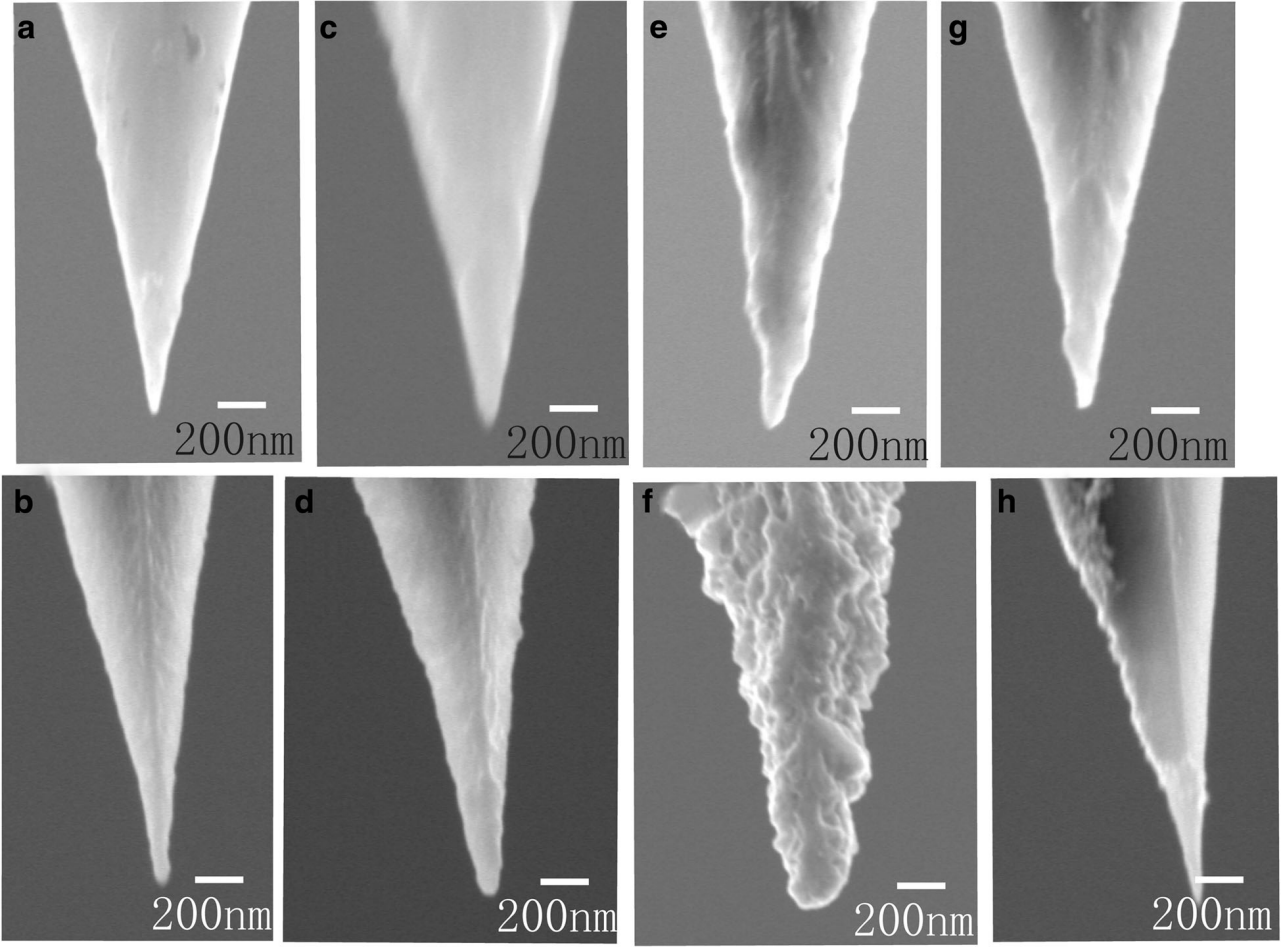
SEM image of probes prepared by wet-chemical procedures. **a** Immersion time 10 min, 1 cycle. **b** Immersion time 5 min, 2 cycles. **c** Immersion time 15 min, 1 cycle. **d** Immersion time 5 min, 3 cycles. **e** Immersion time 30 min, 1 cycle. **f** Immersion time 5 min, 6 cycles. **g** Immersion time 10 min, 1 cycle; the probe apex was damaged during the experiment. **h** Immersion time 10 min, 1 cycle; the probe was not pre-hydroxylated

In our experiment, the probe in Fig. 1d was prepared by immersing the silanized tip in 1.0% HAuCl_4_ and 1.0% NaBH_4_ aqueous solutions for 5 min in turn. Furthermore, two methods were applied to form a compact film on the probe surface. In the first one, the immersion time of HAuCl_4_ varied from 10 min and 15 min to 30 min and then followed by 5 min immersion time in 1.0% NaBH_4_ aqueous solutions. The second experimental path was that we took the process of immersing the silanized tip in 1.0% HAuCl_4_ and 1.0% NaBH_4_ aqueous solutions for 5 min in turn as a cycle and then repeat the above cycle from two and three to six times. Figure 2 shows the Au@AFM probes with different apex diameters via these two improved ways. The probes in Fig. 2a, c, e were prepared with an immersion time of 10 min, 15 min, and 30 min, and their corresponding apex diameters were about 30 nm, 50 nm, and 60 nm, respectively. This indicates that the tip apexes became larger when extending immersion time of silanized tip in HAuCl_4_ aqueous solution. Meanwhile, the tip size increased slowly after 15 min immersion. This can be explained by the fact that MPTS not only serves as a stabilizing agent between the Si probe surface and Au layer but also acts as the primary reducing agent of Au^3+^ ions in wet-chemical reactions. With the increasing diameter, the uncovered MPTS molecules on the probe surface became fewer and fewer, which led to a decreasing quantity of Au^3+^ reduced. In these experiments, the tip size was found to be nearly invariable with time when the immersion time was over 30 min, which showed that the probe was completely covered by Au film after soaking in HAuCl_4_ solution for over 30 min.

In the second improved method of changing the times of immersion cycle, three probes were treated with 5-min immersion time and the cycle number at 2, 3, and 6. The SEM characterization results were given in Fig. 2b, d, f. Here, the total immersion times of the three probes were 10 min, 15 min, and 30 min, which were corresponding to the immersion times of the other three probes in Fig. 2a, c, e. However, the apex diameters of these three probes produced by the second method were larger than those made by the former. This is because the immersion process produces a liquid layer on the probe surface and accordingly forms a new Au film on the probe surface. If the immersion process is repeated, new Au film can be formed on the previous one, so the total thickness of metal film would gradually be increased and the geometric profile of the latter film would be influenced by the former film. Therefore, the probe surfaces prepared by the second method were rougher compared with those prepared by the first method. AFM-TERS probes, which are expensive, are well known for easily losing activity due to the wearing of the thin metal film. Since the second method permits a new Au film formed on the previous one, it is highly possible to make a wear probe recycled, and thus, the cost of TERS could be reduced.

In order to make a convenient comparison, the probes and the corresponding experimental conditions are shown in Table 1.

**Table 1:** The SEM images of probes and corresponding experimental conditions

Probe immersion time	Au deposition time (min)	Time of immersion cycle	Total time (min)
Fig. 2a (30 nm)	10	1	10
Fig. 2b (40 nm)	5	2	10
Fig. 2c (50 nm)	15	1	15
Fig. 2d (75 nm)	5	3	15
Fig. 2e (60 nm)	30	1	30
Fig. 2f (150 nm)	5	6	30

Additionally, the AFM probe is fragile and can easily be damaged during the experiment. The probe shown in Fig. 2g had the same preparation process as that in Fig. 2a (immersed for 10 min in the two solutions), but its diameter was similar to that of the probe in Fig. 2c. This was because the apex of the probe shown in Fig. 2g dropped out for some reason and a flatter apex observed. In order to investigate the TERS enhancement effect of the probes with different apexes, this kind of probes was measured and compared with others in the “TERS of NB” section.

It is worth mentioning that the hydroxylating step for commercial AFM probe was vital during the fabrication of these probes. In another experiment, the hydroxylation step was not included and the probe directly silanized and immersed for 10 min into 1.0% HAuCl_4_ and then 1.0% NaBH_4_ aqueous solutions. The apex diameter of modified tips showed no obvious changes, and some Au nanoparticles were aggregated on the probe surface (Fig. 2h). This occurred because the lack of hydroxylation caused the MPTS to be unevenly adsorbed onto the probe surface, which led to Au nanoparticle aggregation.

### TERS of NB

**Fig. 3:**
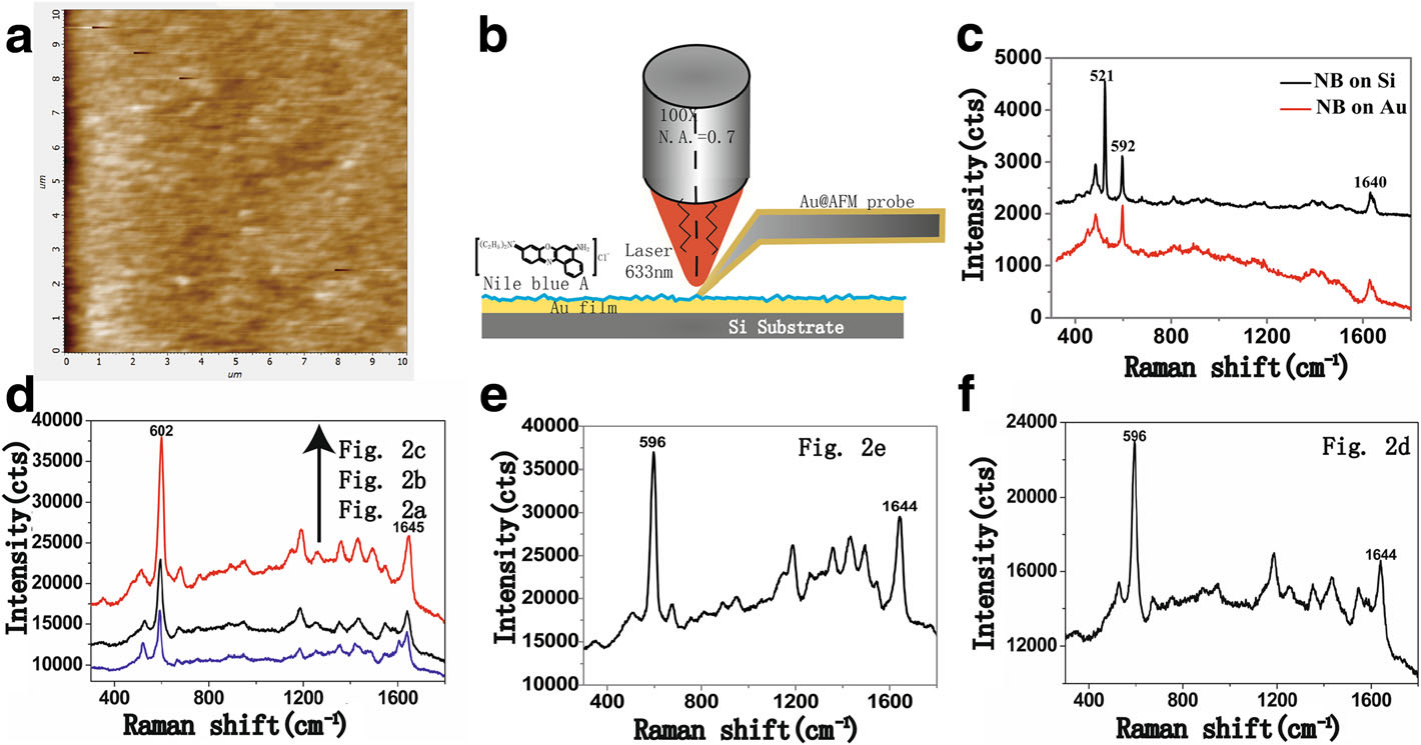
**a** The AFM image of Au substrate. **b** Schematic diagram of a TERS experiment. **c** Raman spectrum of Nile blue (NB) monolayer with tip retracted. **d** Raman spectrum detected by the probes in Fig. 2,a–c. **e** Raman spectrum detected by the probe in Fig. 2e. **f** Raman spectrum detected by the probe in Fig. 2d

SEM images can only provide the thickness of the coating layer on the probe. In order to determine the TERS activity of our as-prepared probe, TERS enhancing performance of the probes should be detected. According to the calculation results of finite-difference time-domain (FDTD), the factors which can influence the TERS signal of a sample are not only probe but the substrate beneath the probe [27]. A metal substrate such as Au, Ag, or Cu will arise a stronger field enhancement owning to the sandwich-type assay called “gap mode.” Therefore, 50 nm Au film was chosen in our experiments as the substrate to test the TERS activities of the probes in Fig. 2. The AFM image of the Au substrate was shown in Fig. 3a. According to the image, the film was smooth, and its surface roughness was less than 3 nm.

The schematic of a TERS experiment was shown in Fig. 3b, in which AFM-TERS feedback with top illumination was used. In this mode, the end of TERS probe was illuminated and efficiently enhanced using an objective lens (× 100, N.A. = 0.7) above the sample. Shadowing effect by the cantilever was avoided using a top visual cantilever. The laser employed was operating at 633 nm wavelength and 5 mW output, and the integration time of Raman signal was 0.1 s. A series of Raman spectra obtained by the system with the present Au@AFM probes was given in Fig. 3d–f.

Before the TERS measurement, we first obtained the Raman signal of NB on Si substrate and the abovementioned Au substrate. As shown in Fig. 3c, except for the Raman peak of Si at 521 cm^−1^, two peaks at 592 cm^−1^ and 1640 cm^−1^ corresponding to the positively charged nitrogen of NB molecules have similar intensity. The result showed that the Au substrate could not enhance the signal of the sample by itself. In the TERS measurement, when the probe was in contact with the sample surface, in addition to the above peaks, peaks of ν_C-N_ (1361 cm^−1^) and ν_C=N_ mode associated to quinoid units (1432 cm^−1^ and 1495 cm^−1^) were detected, and the peak intensity of 592 cm^−1^ increased greatly (see Fig. 3d). The change of the spectral vibration position (592 cm^−1^→602 cm^−1^) was a behavior which has been observed for a long time in near-field Raman spectra tests and ascribed to chemical enhancement by dye-Au charge transfer [28, 29]. These spectral curves indicated that our as-fabricated AFM-TERS probes exhibited Raman enhancement effects. The enhanced Raman scattering of the 592 cm^−1^ peak with the probe in Fig. 2a was about 7 times relative to the signal with the tip retracted. The enhancement is about 12.5 times with the probe in Fig. 2b and 25 for the probe in Fig. 2c. These results were calculated according to the Raman spectra in Fig. 3c, d. This indicated that the peak intensity increased with apex enlargement when the size of the probe apex was less than 50 nm.

The Raman spectrum obtained using the probe in Fig. 2e (~ 60 nm) showed some differences from the one obtained with the probe in Fig. 2c (~ 50 nm, Fig. 3e). However, the intensities of the peaks at 592 cm^−1^ were the same. The Raman spectrum obtained using the probe in Fig. 2d (~ 75 nm) showed that this peak intensity had begun to decrease (Fig. 3f). Using the probe in Fig. 2f (~ 150 nm), the Raman spectrum was not enhanced.

**Fig. 4:**
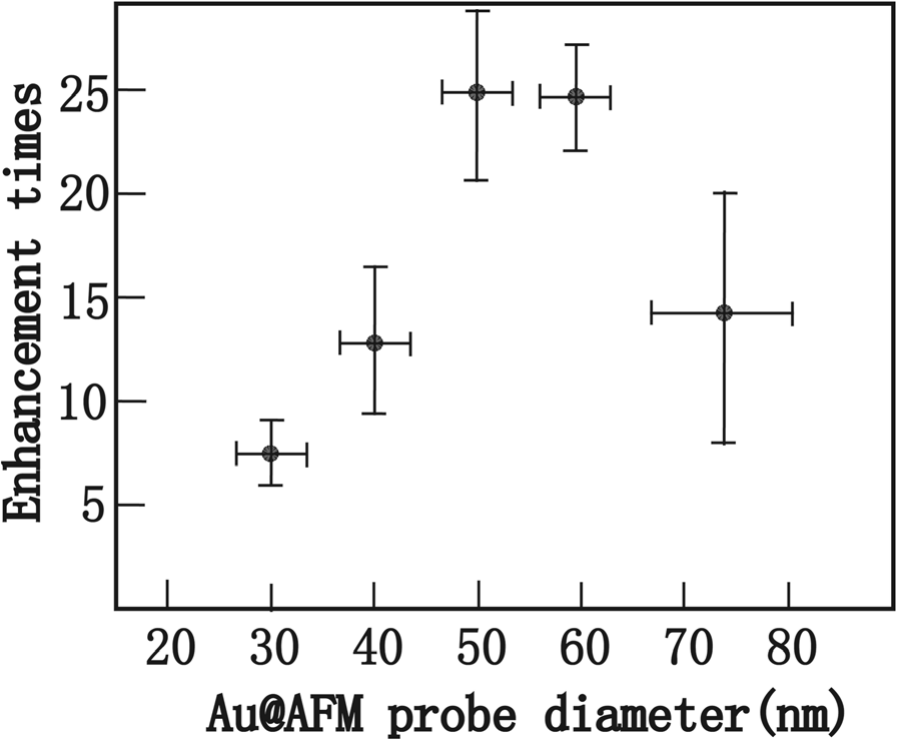
Change of TERS enhancement with the increased diameter of Au@AFM probe

To confirm that the Au@AFM probe prepared by the wet-chemical procedure acted as an effective near-field light source for TERS, ten probes were prepared for every preparation condition according to the probes of Fig. 2a–e respectively. The results of Raman enhancement showed that the enhancement times increase sharply with the increased size of the probe from 30 to 50 nm, and the strongest Raman signal can be achieved when the tip diameter is in the range of 50–60 nm. After 70 nm, the Raman intensity begins to reduce. However, Ren’s group found that the optimized thickness of Au layer was about 60–75 nm, and the theory agreed with the experimental result [30, 31]. According to their calculation model, the tip was regarded as a conical taper terminated by a hemisphere of various radii. Au nanometer thin film on the probe in their experiment followed the Volmer-Weber (VW) mode. So, the computational model was similar to the real probe. For every probe in our experiments, the surface was relatively smooth, and the probe shape is conical-like rather than a hemisphere. Therefore, the discrepancy of the optimized thickness of the Au layers between Ren’s group and ours could be attributed to the shape of the probes. In further experiments, the probes with the apex diameter of 100 nm, 130 nm, and 160 nm were prepared. We found that when the tip diameter was beyond 130 nm, the Raman signal of NB monolayer was no more enhanced. In addition, there was another interesting phenomenon observed in our experiment. The probe in Fig. 2g had the same preparation process as that in Fig. 2a, but it had a similar diameter to the probe in Fig. 2c. The Raman results showed that this probe has a similar enhancement to the probe in Fig. 2c. The result demonstrated that the enhancement effect was independent of Au film thickness; it was related to apex diameter of the probe. The intuitive relationship between the apex diameter and Raman intensity was shown in Fig. 4.

The performance of these fabricated probes was also explored by calculating their Raman enhancement factor (EF) according to the following equation [32]:

$$ \mathrm{EF}\approx \left(\frac{I_{\mathrm{tip}\hbox{-} \mathrm{in}}}{I_{\mathrm{tip}\hbox{-} \mathrm{in}}}-1\right)\frac{A_{\mathrm{FF}}}{A_{\mathrm{NF}}} $$

Where *I*_tip-in_ and *I*_tip-out_ are the Raman peak intensities measured with the probe engaged and disengaged, respectively. *A*_FF_ is the total focal area of the laser, with *A*_FF_ *= πr*_laser_^2^ in which *r*_laser_ = 800 nm. *A*_NF_ is the effective area of the TERS spot, which was estimated according to the apex diameter of AFM-TERS probes and usually obtained by *A*_NF_ ≈ *πr*_tip_^2^. Here, the EF data were calculated according to the intensity of the 592 cm^−1^ peak, which belonged to the vibrational mode of positively charged nitrogen. As for the three Au@AFM probes in Fig. 2b–d, the EFs were 1.5 × 10^3^, 2.9 × 10^3^, and 6.1 × 10^3^, respectively, which indicated that probes with appropriate tip apex diameters exhibited higher Raman enhancement factors. More importantly, the apex diameters were efficiently controlled by the present wet-chemical method, opening a pathway for studying the relationship of the Raman enhancement effect and probe apex diameter.

## Conclusions

In summary, novel AFM-TERS probes were fabricated via a wet-chemical procedure in which MPTS acted both as the reducing agent of Au^3+^ ions and a stabilizing ligand for forming Au@AFM probes. These probes, with appropriate apex size, exhibited dramatic Raman enhancement effects. Importantly, this wet-chemistry procedure possessed characteristics of low cost, simple procedure, high size and shape control, high yield, and universal applicability to Ag and other metallic layers. Also, these probes possessed the advantage of detecting samples in liquid conditions [33–35]. As a metal film produced by physisorption, such as a metal film produced by vacuum evaporation, can peel off in liquid, this situation can be avoided if experimental probes are made by the wet-chemistry procedure, because the metal film and probe were linked by covalent bonds of S–Au.

## Abbreviations

AFM-TERS: Atomic force microscopy-based TERS
Au@AFM probe: Au-coated AFM probe
EDS: Energy dispersive spectrometer
EF: Raman enhancement factor
MPTS: 3-Merraptnpropylt rimethnxysilane
NB: Nile blue
TERS: Tip-enhanced Raman spectroscopy
